# Ten‐Year Results of a Prospective Study on One‐Piece Zirconia Oral Implants for Single‐Tooth Reconstruction

**DOI:** 10.1111/clr.70049

**Published:** 2025-10-02

**Authors:** Ralf‐Joachim Kohal, Agneta Lith, Futoshi Komine, Junichi Honda, Benedikt C. Spies, Felix Burkhardt, Kirstin Vach

**Affiliations:** ^1^ Medical Center – University of Freiburg, Center for Dental Medicine, Department of Prosthetic Dentistry, Faculty of Medicine University of Freiburg Freiburg Germany; ^2^ Department of Oral and Maxillofacial Radiology, Institute of Odontology, Sahlgrenska Academy Gothenburg University Göteborg Sweden; ^3^ Department of Fixed Prosthodontics Nihon University School of Dentistry Chiyoda‐Ku Tokyo Japan; ^4^ Medical Center – University of Freiburg Institute of Medical Biometry and Statistics Freiburg Germany

**Keywords:** clinical investigation, fixed dental prosthesis, oral implant, prospective, zirconia implant

## Abstract

**Objectives:**

The intention of this 10‐year prospective cohort investigation was to clinically and radiographically investigate the outcomes of a one‐piece zirconia implant system for single‐tooth replacement.

**Materials and Methods:**

A total of 65 patients received 66 single‐tooth implants restored with all‐ceramic crowns. Follow‐up assessments were conducted at the time of prosthetic delivery, after 1, 3, 5, and 10 years. Peri‐implant soft tissue parameters were evaluated. To assess peri‐implant bone loss, standardized radiographs were taken at implant placement, prosthetic delivery, at the 1‐, 3‐, 5‐, and 10‐year follow‐ups. Statistical analysis was conducted using linear mixed regression models and Wilcoxon Signed Rank tests to compare differences over time and between groups (*p* < 0.05).

**Results:**

Over the course of 10 years, 16 implants were lost, yielding a cumulative survival rate of 73.3%. Fifteen patients were lost to follow‐up over the 10‐year period. The average marginal bone loss of the remaining implants was 1.09 mm. Probing depth, attachment level, and bleeding showed an increase over time, while plaque index slightly decreased.

**Conclusions:**

Given the high implant loss rate after 10 years and the high occurrence of advanced bone loss observed in this study, the implant was not recommended for routine clinical use.

## 1 Introduction

The clinical use of zirconia implants has seen significant growth over the past decade (Mohseni et al. 2023). They are often viewed as a complement to titanium implants (Kohal and Dennison 2020), which continue to be the gold standard in oral implantology (Sanz et al. 2019). Reasons for choosing zirconia implants include patients' desire for metal‐free restorations, potential hypersensitivity to titanium, or aesthetic concerns when titanium may not be suitable for certain cases (Sicilia et al. 2008; Tschernitschek et al. 2005). Especially, titanium particles/ions released from corroding or wear‐debris‐generating titanium implants have been shown to influence the immune environment of peri‐implant tissues. In particular, they can induce macrophage activation and polarization, often shifting them toward a pro‐inflammatory M1 phenotype. This activation leads to the release of cytokines such as TNF‐α, IL‐1β, and IL‐6, which can impair osteoblastic differentiation and promote bone resorption. Moreover, Ti ions may cause oxidative stress and stimulate the NLRP3 inflammasome, which has been linked to chronic inflammation and peri‐implant bone loss (Huang et al. 2024; Lee et al. 2013; Mombelli et al. 2018; Pettersson et al. 2017). Therefore, an advantage of zirconia implants is the absence of corrosive products, such as titanium particles or ions, which could potentially pose health risks (Hosoki et al. 2016) or contribute to the progression of peri‐implantitis (Fretwurst et al. 2018). Zirconia ceramics resemble the color of natural teeth and offer favorable esthetic properties. Their biocompatibility, e.g., osseointegration, has been demonstrated in various animal studies (Pieralli et al. 2018). In this regard, comparable to titanium implants, zirconia implants with a micro‐rough surface texture are thought to show a higher osseointegration than those with a smooth surface (Altmann et al. 2017; Sennerby et al. 2005). Pre‐clinical studies have also shown that zirconia implant systems can withstand masticatory forces in an artificial oral environment (Bethke et al. 2020; Kohal et al. 2024).

Zirconia possesses good physicochemical characteristics in part due to the high density of 6 g/cm^3^, 0% open porosity, and small grain size (0.35 μm), including a high flexural strength (1200 MPa), Vickers hardness (1200), and fracture toughness K_1c_ (8 MPa·m^1/2^) (Piconi and Maccauro 1999). A mechanism known as transformation toughening is considered to be the basis for the high strength of yttria‐tetragonal ZrO_2_ polycrystal (Y‐TZP). Transformation toughening refers to a toughening mechanism where stress at a crack tip induces a phase transformation from the metastable tetragonal to the monoclinic crystal structure. This transformation is accompanied by a ~4% volume expansion, which exerts compressive stress at a crack tip and impedes crack propagation. As a result, zirconia exhibits significantly improved fracture toughness compared to other ceramics (Chevalier and Gremillard 2009). Given these positive mechanical properties and the toughening effect of phase transformation, zirconia implants made from 3Y‐TZP demonstrate sufficient structural stability for reliable clinical use.

One drawback of zirconia is that it has a tendency to undergo low thermal degradation (Chevalier and Gremillard 2009), and it remains unclear whether this affects the long‐term stability and success of zirconia as an implant material. Nevertheless, clinical outcomes of zirconia implants are reported to be comparable to those of titanium implants in mid‐term studies with observation periods of up to 5 and 10 years (Mohseni et al. 2023). However, long‐term studies with follow‐up periods of 10 years or more are scarce (Mohseni et al. 2023). Therefore, this prospective clinical cohort study aimed to assess the survival rate and marginal bone remodeling of a one‐piece zirconia oral implant used for single tooth replacement. The present paper presents the 10‐year data of the zirconia implant system.

## 2 Materials and Methods

### 2.1 Study Population, Investigated Implant System, and Surgical Procedure

Patients aged between 18 and 70 years without systemic diseases who required single‐tooth replacement were recruited for this study. Exclusion criteria included general health conditions and alcohol or drug abuse that contraindicated surgical procedures. Local contraindications comprised conditions such as tumors and ulcers (for more information see (Kohal et al. 2018)). All participants provided written informed consent, and the study protocol was approved by the local ethics committee (Investigation Number: 337/04 and 337/04_170759; University Medical Center Freiburg, Freiburg, Germany) and followed the STROBE guidelines. The study conforms to the Declaration of Helsinki—Ethical Principles for Medical Research Involving Human Participants adopted by the 18th WMA General Assembly, Helsinki, Finland, June 1964 and amended by the 75th WMA General Assembly, Helsinki, Finland, October 2024.

The surgical procedure was described in detail elsewhere (Kohal et al. 2018). In this study, a conical, one‐piece implant system made of 3% mol yttria‐stabilized tetragonal zirconia polycrystal (3Y‐TZP) with a moderately (Sa = 1.24 μm) rough porous surface (ZiUnite, Nobel Biocare AB, Gothenburg, Sweden) was applied. Implants were placed immediately after tooth extraction or in healed sites (flapless; using a tissue punch; full‐thickness flap).

Standardized radiographs were taken using intraoral X‐ray film holders customized to the intraoral situation. After implant placement, marginal bone levels were evaluated using standardized radiographs. The implant abutments were cut using slight chamfer preparations with new torpedo‐shaped cylindrical diamond burs (medium and fine grit: 107 μm/46 μm; Brasseler Komet, Lemgo, Germany), under continuous water cooling. Approximately 6 weeks after implant placement in the mandible and 14 weeks in the maxilla, impressions were taken. Prior to impression taking, retraction cords were placed, and custom trays fabricated by a dental technician were used with a polyether impression material (Impregum, 3M Espe, Seefeld, Germany). Impressions of the opposing dentition were made with alginate using rigid, prefabricated stainless‐steel trays, and centric relation was recorded with a medium‐viscosity A‐silicone bite registration material (Greenbite Fast‐Set, Detax, Ettlingen, Germany). All prosthetic work was performed by a single dental laboratory accredited by the material manufacturer. Working models were poured, scanned, and digitized using a tactile scanner (Procera Forte, Nobel Biocare). The digital data were then imported into the Procera CAD software for framework design. Subsequent milling of presintered zirconia blanks (Procera Zirconia, Nobel Biocare) was carried out at a centralized production facility in Sweden using a computer‐aided manufacturing (CAM) process. Upon delivery, each framework required manual adjustment by the dental technician to ensure precise fit. Following successful intraoral try‐in, the frameworks were returned to the laboratory for finalization with hand‐layered veneering using a silicate ceramic (NobelRondo Zirconia, Nobel Biocare). All single crowns were definitively cemented with a glass ionomer cement (Ketac Cem, 3M Espe; Neuss Germany). Occlusion was carefully verified using 12 μm articulating foil and 8 μm shimstock foil, both on the restorations and the remaining dentition, and adjustments were made where needed using fine and extra‐fine diamond burs (46 μm/25 μm) followed by a three‐step polishing system for ceramics (Brasseler Komet). Final treatment included detailed oral hygiene instructions for all patients at the time of crown delivery. The definitive single crown (SC) restoration was delivered after a healing period of approximately 2 months for mandibular and 4 months for maxillary implants. Any remaining cement was meticulously removed, and patients were scheduled for a follow‐up assessment 1–3 days after cementation.

### 2.2 Removal of Failing Implants

To remove failing implants, full‐thickness mucoperiosteal flaps were elevated around each site. In order to improve visibility of the surrounding bone, inflammatory soft tissue adjacent to the implants was detached using a periodontal scaler. Bone apical to the immobile implants was then circumferentially resected to the level of the implant apex using a trephine bur with an inner diameter slightly larger than the implant's outer diameter (4.3 or 5 mm), under copious irrigation. The depth of resection was guided by the known implant lengths (10, 13, or 16 mm). In cases where the implants remained immobile after trephination, a raspatory was used to gently fracture the residual bone beneath the apex and facilitate implant removal.

### 2.3 Radiographical and Clinical Assessment and Follow‐Ups

Throughout the investigation, the patients participated in an annual maintenance program, which included regular check‐ups, oral hygiene instruction, and professional cleanings.

Patients underwent follow‐up evaluations at 1, 3, 5, and 10 years with assessment of probing depth (PD), clinical attachment loss (CAL), bleeding on probing (BOP), and plaque index (PlI) at teeth and implants. To track bone remodeling over time, x‐rays were taken in the long‐cone parallel technique at implant placement (baseline; IP), prosthetic insertion (PI), and at 1‐year, 3‐year, 5‐year, and 10‐year follow‐ups. An independent radiologist (A.L.) analyzed all radiographs.

Based on the following criteria, implant success at the 10‐year follow‐up was determined: PD of ≤ 5 mm, no bleeding on probing, absence of suppuration, and a maximum bone loss of ≤ 2 mm—accounting for an expected loss of 1 mm in the first year, followed by an annual loss not exceeding 0.1 mm. Peri‐implantitis was diagnosed in cases where bleeding on probing or suppuration was present, the probing depth was ≥ 6 mm, and bone loss exceeded 2 mm (see: Berglundh et al. 2018; Karoussis et al. 2003; Schwarz et al. 2018). The criteria from van Steenberghe (1997) for implant success were applied.

### 2.4 Statistical Analysis

Descriptive statistics are presented as means with standard deviations. With an actuarial life table analysis (Altman 1999), the cumulative survival rates of zirconia implants were determined. To assess influencing factors for changes in bone levels, PD, CAL, and linear regression models were used, adjusting for age and gender. For time comparisons, corresponding linear mixed models were used. Pairwise comparisons were made, with adjustments for multiple testing using the Scheffé method. Due to their non‐normal distribution, Wilcoxon Signed Rank tests were performed to compare BOP and PlI value differences between teeth and implants. Using the Kaplan–Meier method, implant survival rates were estimated. Group comparisons were made with Cox proportional hazards models. For the parameter most indicative of implantation success, marginal bone loss, results were verified using multiple imputation methods (10 imputations). A significance level of 0.05 was applied to all statistical tests. All statistical analyses were conducted using STATA version 17.0 (StataCorp, College Station, Texas, USA).

## 3 Results

A total of 65 patients (40 females) were treated with 66 single‐tooth implants; one patient received two single‐tooth implants. Due to early implant loss (three implants were lost 1–3 months after implant insertion), only 62 patients (63 implants) received their permanent single crown(s).

Ten patients lost their replaced teeth due to periodontitis and the remaining 55 patients lost theirs due to caries. Only two patients reported serious illnesses (diabetes, fibromyalgia), while the others did not. Three patients were undergoing treatment for hypothyroidism and one patient received medication (oral antidiabetic agent) for type 2 diabetes, which was well controlled. In addition, four patients were smokers and four further patients showed signs of bruxism.

The mean age of the patients was 40.4 ± 13.0 years. The distribution of the zirconia oral implants regarding upper and lower jaw, implant diameter, and implant loss can be depicted from Table 1.

**TABLE 1:** Implant dimensions, implant area, and implant failure after 10 years.

Platform [Ø mm]	Length [mm]	Upper jaw		Lower jaw	
		Placed	Failed	Placed	Failed
RP 4.3	10	1	0	10	3
	13	1	0	6	2
	16	3	0	0	0
	Total	5	0	16	5
WP 5.0	10	1	0	9	4
	13	9	2	18	5
	16	3	0	5	0
	Total	13	2	32	9

Abbreviations: RP, regular platform; WP, wide platform.

A total of 61 implants were placed in healed sites, while only 5 implants were placed in extraction sockets (Table 2). The majority of surgeries involved raising flaps without releasing incisions (36 cases) or performing flapless surgery using a punch (18 cases). Regarding the primary stability applying insertion torque, 4 implants showed torque values below 35 Ncm, 38 implants ranged between 35 and 45 Ncm, and 17 implants exhibited insertion torques exceeding 45 Ncm. For the remainder of implants, no torque value was registered.

**TABLE 2:** Different operating factors used in the present investigation.

	Periodontitis	Caries	Trauma	Immediate	Late	Flaps without releasing incision	Flaps with releasing incision	Punch technique	Flapless/extraction sockets
Cause of extraction	10	55 patients 56 teeth	0						
Time of implant placement				5	61				
Surgical procedure						36	7	18	5

Most implant sites demonstrated a bone quantity of B and a quality of II (Lekholm and Zarb 1985).

### 3.1 Implant Survival

Sixty‐three out of 66 implants (62 patients) were restored with permanent all‐ceramic crowns since three implants (upper and lower premolar, upper molar) did not integrate and were lost before restoration (Table 3). Of the 62 patients who were restored with SC, 61 attended the 1‐year follow‐up. One patient, due to business reasons, relocated and could no longer be contacted, thus was counted as a drop‐out before 1 year. Between the 1‐year and 3‐year follow‐ups, three implants (replacing two mandibular molars and a mandibular premolar) were lost due to increased peri‐implant bone loss. At the 3‐year follow‐up, two additional patients were considered drop‐outs: one missed this and following follow‐ups due to scheduling conflicts, and the other moved without providing a new address. As a result, 56 patients were evaluated at the 3‐year follow‐up. At the 5‐year follow‐up, 48 patients were evaluated, with one more patient relocating and becoming unreachable. Between the 3‐year and 5‐year follow‐ups, seven implants (one lower premolar and six lower molars) were lost. Six implants were removed due to peri‐implant infection, and one fractured, although it had a history of peri‐implantitis. At the 10‐year follow‐up, 35 patients with 35 implants were seen. Between the 5‐year and 10‐year follow‐ups, three more implants were lost in two patients (one patient lost both implants) due to peri‐implantitis leading to 16 lost implants over the 10‐year period. Furthermore, 11 patients refused to participate at the 10 year follow‐up or did not react to the follow‐up invitation, counting as drop‐outs.

**TABLE 3:** Patient enrollment, implant losses, patient drop‐outs and final outcome.

Patient enrollment, implant losses, and patient drop‐outs and final outcome							
	Patients	Implants	Before PrD	PrD—1‐year FUP	1‐year FUP—3‐year FUP	3‐year FUP—5‐year FUP	5‐year FUP—10‐year FUP
Enrollment	65	66					
Implant loss			3				
Prosthetic delivery	62	63					
Drop‐out				1			
1‐year FUP	61	62					
Drop‐out					2		
Implant loss					3		
3‐year FUP	56	57					
Drop‐out						1	
Implant loss						7	
5‐year FUP	48	49					
Drop‐out							11
Implant loss							3 in 2 patients
10‐year FUP	35	35					

Abbreviations: FUP, follow‐up; PrD, prosthetic delivery.

**FIGURE 1:**
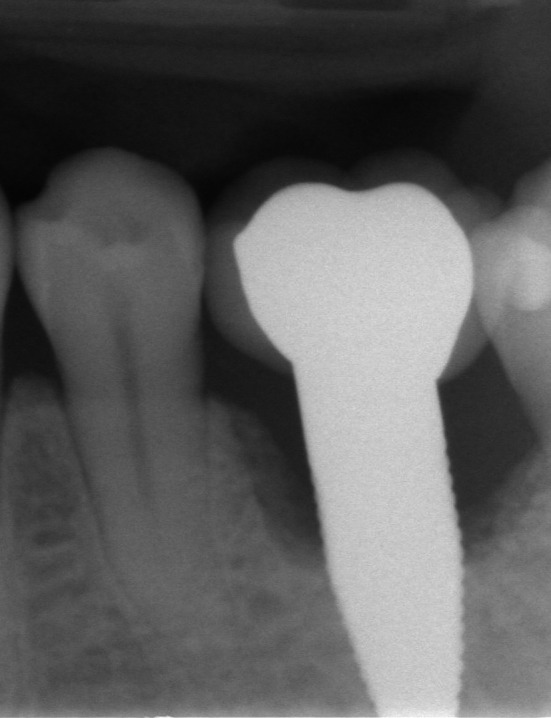
Failing implant in the position of the 1st molar which was removed between the 5‐ and 10‐year follow‐up showing an extended peri‐implant bone loss.

**FIGURE 2:**
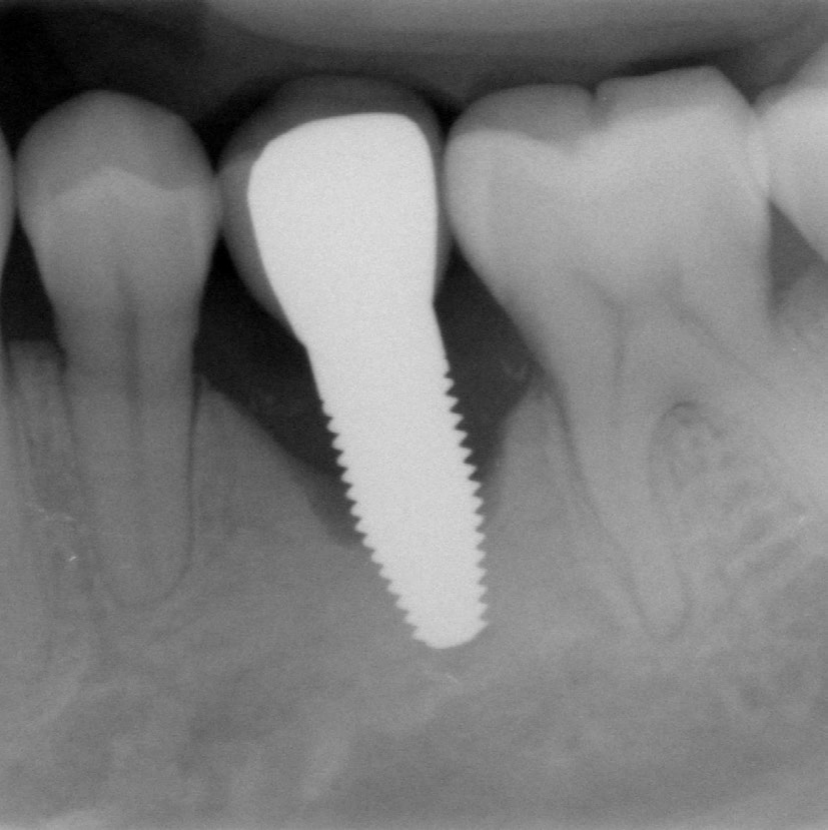
Failing implant in the position of the 2nd premolar which had to be removed between the 5‐ and 10‐year follow‐up with bone loss up to the tapered implant tip.

**FIGURE 3:**
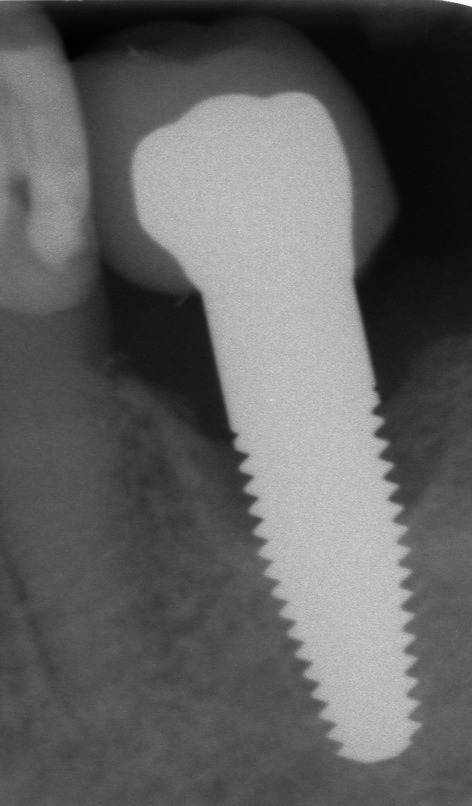
Failing implant in the 1st molar position. The radiograph shows bone loss until the transition between the straigth and tapered endosseous implant area. This implant was removed between the 5‐ and 10‐year follow‐up.

**FIGURE 4:**
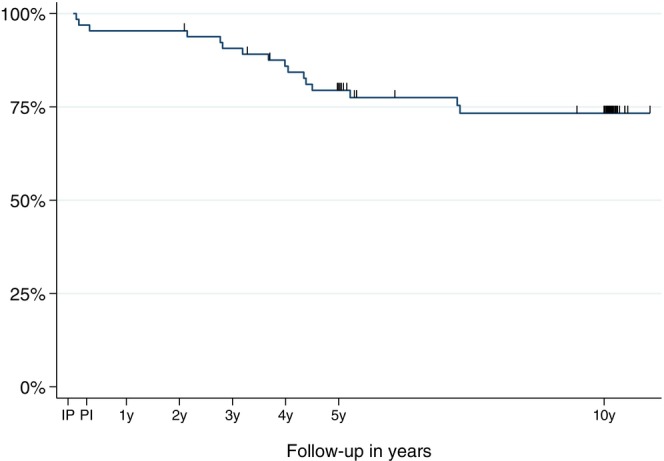
Kaplan–Meier survival estimate of the implants from implant placement (IP) to the 10‐year follow‐up (10 y) (IP, implant placement; PI, prosthesis insertion).

The calculated 10‐year implant survival rate was 73.31% [CI: 60.0%–82.8%] (Figures 1, 2, 3, 4). Implant length exhibited a significantly (*p* < 0.001) higher survival rate for 16 mm implants (100%) compared to 13 mm (70.39%, 95% CI 50.0%–83.7%) and 10 mm (65.34%, 95% CI 40.7%–81.8%) implants.

### 3.2 Marginal Bone Loss and Overall Implant Success

**FIGURE 5:**
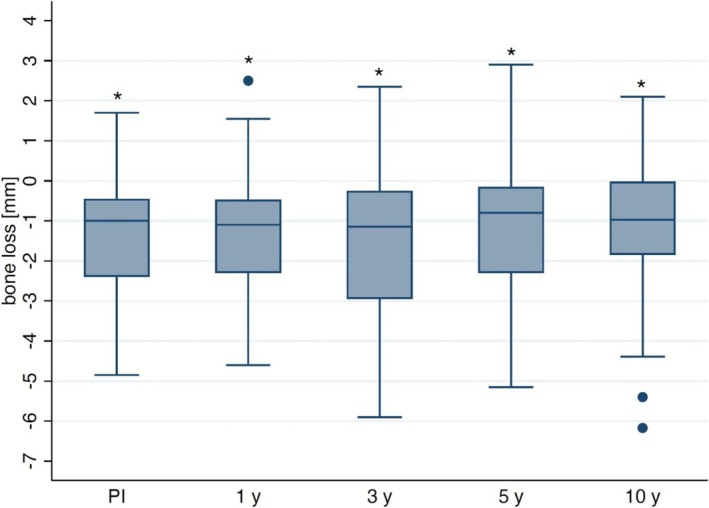
Boxplots showing bone loss (in mm) at prosthesis insertion (PI), at the 1‐year (1 y), 3‐year (3 y), 5‐year (5 y), and 10‐year (10 y) follow‐up in comparison to implant placement (IP). Asteriks (*) indicate significances (*p* < 0.001) compared to IP.

An average bone loss of 1.09 ± 1.89 mm was shown at the 10‐year follow‐up when the standardized radiographs of the remaining implants were evaluated (Figure 5), representing a significant increase in bone loss compared to the time of implant placement (IP) (difference: 1.32 ± 1.47 mm, *p* < 0.001). In comparison, the mean bone loss relative to IP was 1.31 ± 1.50 mm at the 1‐year follow‐up and 1.46 ± 1.95 mm and 1.09 ± 1.84 mm at the 3‐year and 5‐year follow‐ups, respectively. Radiographic evaluations were possible for 57 implants at the 1‐year follow‐up, for 56 implants at 3 years, and for 42 implants at 5 years. However, only 34 implants were available for radiographic assessment at the 10‐year follow‐up. At the 10‐year follow‐up, bone changes varied across implants: 8 implants showed bone gain (2 implants more than 2 mm, 2 implants > 1 mm and < 2 mm, 4 implants between 0.02 and 0.59 mm), 10 implants lost ≤ 1 mm of bone, 9 implants lost between > 1 and ≤ 2 mm, 1 implant lost > 2 and ≤ 3 mm (2.4 mm), 3 implants lost > 3 and ≤ 4 mm, 1 implant lost > 4 and ≤ 5 mm, and 2 implants experienced bone loss exceeding 5 mm (5.4 and 6.2 mm). Among the 34 implants assessed radiographically, 7 (20.5%) exhibited marginal bone loss > 2 mm. On the basis of the success criteria for bone loss after 10 years (≤ 2 mm) as defined by Karoussis et al. (2003), the calculated overall implant success rate at the 10‐year follow‐up was 56.75% [41.75%–69.26%].

Implants with 10 mm length showed significantly less bone loss than 13 mm long implants (*p* = 0.039).

Implants with a diameter of 4.3 mm showed less loss (*p* = 0.033) than implants with a diameter of 5 mm.

### 3.3 Clinical Parameters

**FIGURE 6:**
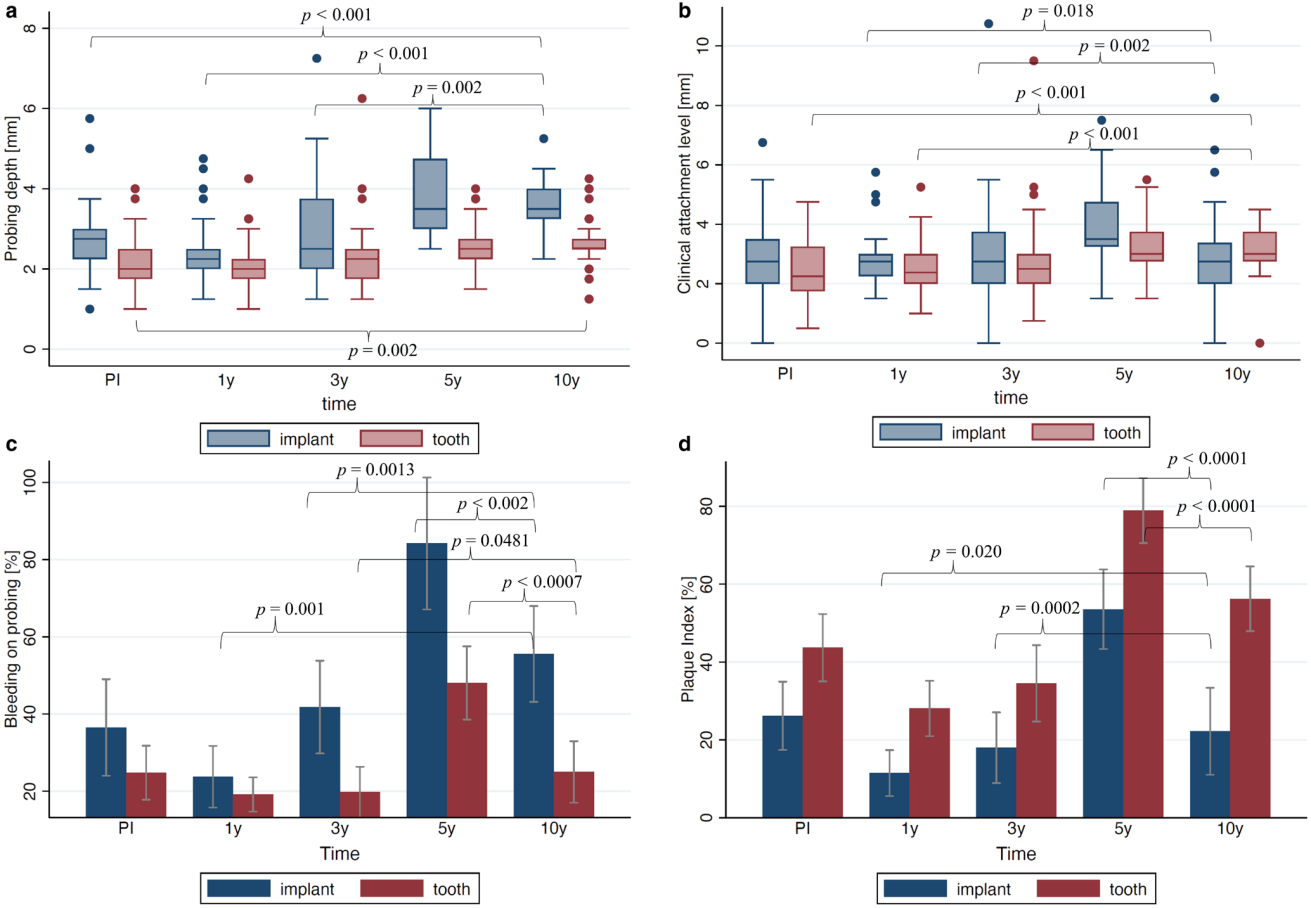
Probing depth (a), clinical attachment loss (b), bleeding on probing (c), and plaque index (d) at prosthesis insertion (PI), 1‐year follow‐up (1 y), 3‐year follow‐up (3 y), 5‐year follow‐up (5 y), and 10‐year follow‐up (10 y). Significant differences (*p* < 0.05) are visualized compared to 10‐year follow‐up.

The mean PD of the implants at the 10‐year follow‐up was 3.54 ± 0.67 mm (Figure 6a, Table 4), which was significantly higher than at PI (2.76 ± 0.75 mm; *p* < 0.001), at the 1‐year follow‐up (2.32 ± 0.68 mm; *p* < 0.001), and at the 3‐year follow‐up (3.03 ± 1.23 mm; *p* = 0.002). Compared to the 5‐year follow‐up, a slight improvement was observed (3.83 ± 1.01 mm; *p* = 0.084). A significant increase in PD from PI to the 10‐year follow‐up was also noted for the reference teeth (*p* < 0.001). The PD increase at the implant sites was significantly greater compared to the reference teeth (*p* = 0.001), regardless of the patients' gender or age. A frequency analysis of PD around implants at the 10‐year follow‐up revealed the following results: 22 implants showed PDs of 4 mm or less, 10 implants had PDs of 5 mm, and 4 implants showed PDs of 6 mm.

The CAL at the implant sites was at the 10‐year follow‐up with a value of 3.04 ± 1.53 mm (Figure 6b, Table 4) higher than at PI (2.90 ± 1.23 mm; *p* = 0.357) and at the 1‐year follow‐up (2.69 ± 0.75 mm; *p* = 0.018). This value was comparable to the 3‐year follow‐up (3.06 ± 1.52 mm; *p* = 0.526) and significantly lower than the 5‐year follow‐up (3.96 ± 1.26 mm; *p* = 0.002). CAL increased significantly at tooth sites between PI and the 10‐year follow‐up (*p* < 0.001). Additionally, significant differences were observed between the 10‐year follow‐up and both the 1‐year and 3‐year follow‐ups (*p* < 0.001) but not to the 5‐year follow‐up (*p* = 0.559). No significant differences in CAL between implant sites and reference teeth were observed from PI at the 10‐year follow‐up (*p* = 0.089).

BOP at the implant sites with 55.56% ± 37.45% (Figure 6c, Table 4) at the 10‐year follow‐up was not significantly increased compared to PI (36.51% ± 50.57%; *p* = 0.137). However, the BOP increased significantly compared to the 1‐year follow‐up (23.77% ± 31.76%; *p* = 0.001) and the 3‐year follow‐up (41.81% ± 46.17%; *p* = 0.001), but decreased significantly compared to the 5‐year follow‐up (84.18% ± 60.96%; *p* = 0.002). The BOP at the reference teeth showed significantly lower changes than at the implant sites (all *p* = 0.006) after 10 years.

**TABLE 4:** Soft‐tissue parameters PD, CAL, BOP, and PlI after PI, at the 1‐year, 3‐year, 5‐year and 10‐year follow‐up, standard deviation in brackets.

		PD [mm]	CAL [mm]	BOP [%]	PlI [%]
PI	Implants	2.76 (0.75)	2.90 (1.23)	36.51 (50.57)	26.19 (35.48)
	Reference teeth	2.10 (0.59)	2.43 (0.99)	24.78 (38.12)	43.70 (47.28)
1 year	Implants	2.32 (0.68)	2.69 (0.75)	23.77 (31.76)	11.48 (23.53)
	Reference teeth	1.94 (0.48)	2.49 (0.68)	19.15 (24.08)	28.07 (38.81)
3 years	Implants	3.03 (1.23)	3.06 (1.52)	41.81 (46.17)	17.98 (34.56)
	Reference teeth	2.25 (0.65)	2.67 (0.99)	19.76 (34.27)	34.52 (51.12)
5 years	Implants	3.83 (1.01)	3.96 (1.26)	84.18 (60.96)	53.57 (36.44)
	Reference teeth	2.60 (0.49)	3.21 (0.82)	48.06 (45.78)	78.89 (40.35)
10 years	Implants	3.54 (0.67)	3.04 (1.53)	55.56 (37.45)	22.22 (34.21)
	Reference teeth	2.62 (0.54)	3.18 (0.77)	25.00 (32.43)	56.25 (33.92)

Abbreviation: PI, prosthesis insertion.

At the time of prosthesis insertion, the PlI at the implant sites had a value of 26.19% ± 35.48%. A significant reduction at the 1‐year follow‐up (11.48% ± 23.53%; *p* = 0.004) occurred, but no significant reduction at the 3‐year follow‐up (17.98% ± 34.56%; *p* = 0.065) (Figure 6d, Table 4). At the 5‐year follow‐up, the PlI had significantly increased to 53.57% ± 36.44% (*p* = 0.001), but it reduced significantly (*p* = 0.0001) from the 5‐year to the 10‐year follow‐up (22.22% ± 34.21%). Significant differences in PlI changes at the 10‐year follow‐up were observed between the implant sites and the reference teeth compared to PI (*p* = 0.020) and to the 3‐year follow‐up (*p* = 0.001).

### 3.4 Peri‐Implant Health, Mucositis, and Peri‐Implantitis Conditions

The classification criteria described by Berglundh et al. (2018) regarding peri‐implant health, peri‐implant mucositis, and peri‐implantitis for the remaining implants were applied. After 10 years, 16 implants demonstrated peri‐implant health, characterized by the absence of bleeding on probing (BOP), erythema, swelling, or suppuration and bone loss ≤ 2 mm. Thirteen implants showed peri‐implant mucositis, indicated by positive BOP, probing depths (PD) of ≤ 5 mm, and bone loss of ≤ 2 mm. One of those mucositis‐suffering implants had a bone loss of 2.4 mm; however, the radiographic and clinical data did not indicate peri‐implantitis. Six implants suffered from peri‐implantitis, presenting with positive BOP, suppuration, PD ≥ 6 mm, and bone loss exceeding 2 mm.

The infection in the peri‐implant tissues was treated according to the Cumulative Interceptive Supportive Therapy (C.I.S.T.) protocol described by Lang et al. (2004).

### 3.5 Survival and Success of the Implant‐Supported Single Crowns

Among the 35 restorations evaluated after ten years, five crowns required refabrication before the 5‐year follow‐up due to major veneer chipping. No crown had to be replaced thereafter. However, all except four anterior crowns showed major chipping (codes “Charly” and “Delta”) of the ceramic veneering according to the United States Public Health Service (USPHS) criteria (Spies et al. 2019). The survival rate of the SC at the 10‐year follow‐up was 85.7%, but the success rate was only 11.4% (Figures 4, 5, 6).

## 4 Discussion

This prospective case series reports 10‐year outcomes of one‐piece zirconia implants with a moderately rough surface for SC restorations. Of the 66 implants placed, 16 were removed, resulting in a 73% survival rate—lower than the 10‐year survival rates reported for titanium implants (90%–95%) (Hjalmarsson et al. 2016; Howe et al. 2019).

Long‐term data on zirconia oral implants remains limited. Lorenz et al. (2019) evaluated one‐piece zirconia implants restored with SC over an observation period of up to 9.7 years (mean: 7.8 years) with a survival rate of 100%. Brunello et al. (2022) followed 30 patients over a 9‐year period and reported an implant survival rate of 81.7%, since peri‐implantitis was diagnosed in 10 patients. Similar to this study, the primary cause for failure was peri‐implantitis.

The lower mean bone loss observed in the present investigation at the 5‐ and 10‐year (1.09 mm) compared to the 1‐year (1.31 mm) and 3‐year (1.46 mm) follow‐ups is primarily due to the removal of 13 implants with severe bone loss by the 5‐year follow‐up. In contrast, only two additional implants were removed between the 5 and 10‐year follow‐ups. This stability among the remaining implants suggests a more favorable prognosis after 5 years.

Bone loss was observed early in the study without an increase in PD and CAL at the 1‐year follow‐up (Kohal et al. 2012). BOP and PlI showed low values indicating healthy marginal tissues; this may indicate that bone loss is primarily not a result of bacterial colonization and subsequent inflammation but might have been influenced by other factors. One reason for the increased bone loss might have been the entrapment of cement that had to be used to fixate the crowns on the implants. However, meticulous care was taken to remove all remnants at crown insertion and subsequent follow‐up visits.

Similar negative outcomes regarding bone loss have been reported for the NobelDirect titanium implant—which was similar in design to the evaluated zirconia implant—where the observed early bone loss was attributed to its tapered macrodesign. It was discussed that the tapered design exerted physical pressure on the crestal bone (Östman et al. 2007), leading to the observed early bone loss. Additionally, excessive torque during implant insertion might have led to stress concentration in the implant neck area, potentially causing bone damage. Whether insertion torques of 45 Ncm or higher were responsible for bone loss could not be statistically confirmed in a univariate analysis of marginal bone loss from implant insertion to the 5‐ (Kohal et al. 2023) and 10‐year follow‐up.

The etiology of tooth loss—whether due to periodontal disease or caries—may influence peri‐implant healing dynamics, marginal bone remodeling, and overall implant success. Teeth extracted as a result of advanced periodontitis are frequently associated with vertical bone defects, chronic inflammation, and compromised local tissue conditions, which can predispose patients to increased peri‐implant bone loss and even implant failure if there is a continued tendency toward periodontal breakdown (Carra et al. 2023; Orishko et al. 2024). Systematic reviews confirm that individuals with a history of periodontitis face a higher risk of implant failure and significantly greater marginal bone loss compared to periodontally healthy individuals (RR≈1.89; SMD≈0.44) (Serroni et al. 2024; Sgolastra et al. 2015). Comprehensive periodontal therapy and consistent follow‐up care are essential to ensure implant success in such cases.

In contrast, tooth loss resulting from deep caries or endodontic failure is commonly associated with apical pathology or periapical bone destruction, which, while more localized, can also influence early healing outcomes. Apical pathology has been identified as a potential risk factor for retrograde peri‐implantitis, particularly in immediate or early implant placement protocols (Di Murro et al. 2021). Literature describes “endo‐implant defects” (Daubert et al. 2020) arising from persistent periapical infections of adjacent teeth, resulting in localized bone loss at the implant site—often reversible upon successful endodontic treatment or extraction (Gao and Ma 2024).

While the influence of caries‐related extractions on implant success remains less clearly defined, the potential presence of residual apical lesions or subclinical infection suggests that thorough site debridement and infection control remain critical, particularly when immediate implantation is planned (Chrcanovic et al. 2015; Colak and Demirsoy 2023).

The implant's surface design may have contributed to the increased peri‐implant bone loss associated with secondary infection. The ZiUnite zirconia surface was a porous surface, initially developed to enhance bone‐to‐implant contact, demonstrating promising results in animal investigations (Sennerby et al. 2005). Similarly, investigations of retrieved ZiUnite implants reported favorable osseointegration in regions where the porous surface remained in contact with bone, with an average bone‐to‐implant contact of 76.5% (Kohal et al. 2016).

Besides osseointegration, in vivo aging—specifically low‐temperature degradation (LTD)—was identified as a potential factor contributing to bone and subsequent implant loss (Kohal et al. 2025).

The stability of 3 mol% yttria‐stabilized tetragonal zirconia polycrystal (3Y‐TZP) is affected by the inherent metastability of its tetragonal crystal structure at ambient temperatures (Chevalier and Gremillard 2009). While this characteristic enables beneficial toughening through stress‐induced phase transformation, it simultaneously renders the material susceptible to phase changes triggered by exposure to aqueous environments, such as saliva or physiological fluids—a degradation process commonly termed low‐temperature degradation (LTD) or aging. LTD manifests as a gradual surface deterioration resembling stress corrosion, initiated by the infiltration of water molecules that induce roughening of the material's surface and promote the formation of microcracks. This deterioration mechanism presents a significant challenge for the use of yttria‐stabilized zirconia (YSZ) in oral implantology. In this specific case, it appears that the instability within the porous surface layer was pivotal to the failure of the implant. Rapid phase transformation from tetragonal to monoclinic structure, accompanied by microcracking within the porous coating upon exposure to body fluids, resulted in a breakdown and eventual detachment of the porous layer from the core zirconia material. This loss of structural integrity compromised the essential contact between the implant and the surrounding bone, ultimately leading to bone resorption. Furthermore, aging‐induced fragmentation of the porous surface may have released particulate matter, potentially triggering local inflammatory responses similar to those documented for titanium implants (Fage et al. 2016; Mombelli et al. 2018). This inflammatory reaction likely contributed to peri‐implant bone degradation and the eventual failure of osseointegration. In fact, it is plausible that fragmentation of the surface layer began shortly after implantation, releasing pro‐inflammatory debris at an early stage. Moreover, LTD‐related bone loss could have exposed additional porous surface areas, making them more vulnerable to bacterial plaque infiltration and exacerbating peri‐implant tissue inflammation (Al‐Ahmad et al. 2013).

Several studies employing advanced imaging and elemental mapping techniques have documented delamination and zirconium (Zr) migration at the bone–implant interface—parallel phenomena more commonly reported for titanium implants. Investigations utilizing Environmental Scanning Electron Microscopy coupled with Energy Dispersive X‐Ray spectroscopy (ESEM–EDX) found titanium particles were detected not only at the implant surface but also several millimeters into adjacent bone tissue, confirming ion translocation and micro‐migration over time (Gandolfi et al. 2018; Romanos et al. 2022). Similarly, analyses of human implants retrieved after 4 months showed distinct bone mineralization zones through ESEM–EDX; although primarily focused on mineral composition, they set a robust precedent for using elemental science in histological implant assessment (Prati et al. 2020). Most relevantly to zirconia, a failed human zirconia implant retrieved after only 3 weeks was evaluated with VP‐SEM and EDX, detecting early deposition of Zr (along with Ca and P) on the implant threads—evidence of Zr release and interaction with the forming bone matrix (Borie et al. 2024). A study on inflamed human tissues around titanium and zirconia oral implants exhibiting signs of peri‐implantitis evaluated the size, distribution, and chemical speciation of the exogenous micro‐ and nanosized particles using synchrotron μ‐X‐ray fluorescence spectroscopy (XRF), nano‐XRF, and μ‐X‐ray absorption near‐edge structure (XANES) (Nelson et al. 2020). The authors stated that titanium and Y‐TZP debris were identified in the human tissues. The investigation verified that ceramic particles can accumulate in substantial quantities adjacent to zirconia oral implants in human peri‐implant soft tissues. Ti and ceramic implant fragments ranged in size from the microscale to the small nanoscale. Additionally, in a mini‐pig model, laser ablation ICP‐MS analysis revealed that Zr released from zirconia implants accumulates in surrounding maxillary bone tissue at measurable levels (0.59–0.75 mg Zr/kg bone), albeit lower than titanium, yet clearly indicating Zr migration and potential for histological alteration (He et al. 2020). Beyond elemental translocation, micromorphological examinations via backscatter‐SEM on retrieved implants have shown evidence of delamination at the bone–implant interface (Romanos et al. 2022). Together, these micromorphological and elemental findings—obtained through ESEM‐EDX, SEM‐EDX, LA‐ICP‐MS, and VP‐SEM—strongly support the presence of Zr release and interface delamination in both zirconia and titanium implants, warranting further discussion in the context of long‐term implant stability and biocompatibility.

Among the 35 restorations assessed at the 10‐year follow‐up, the survival rate for SC was 86.1%. However, the overall success rate was notably low at 11.1% due to the high incidence of major chipping of the ceramic veneering. At the time of the study conceptualization, all‐ceramic restorations in the posterior region were typically veneered with a ceramic layer (Stawarczyk et al. 2017). Our results are consistent with a recent systematic review that documented a 38% chipping rate in SC restorations during the longest evaluated period of 5 years (Spitznagel et al. 2022).

The lack of a control group presents a limitation of the present study (de Beus et al. 2024). Including a control group with implants that differ in macro‐ and/or micro‐design could help elucidate which implant‐related factors are associated with peri‐implant bone loss. The variability introduced by different implantation time points and non‐standardised surgical techniques further adds to the study's limitations. This variability and the high drop‐out rate furthermore may reduce the generalizability of the results.

Nevertheless, the reporting of such limitations and of unexpected or adverse outcomes plays a critical role in implant research. Negative or non‐confirmatory data are essential for building a complete and unbiased evidence base, especially in clinical fields where positive outcomes are disproportionately represented in the literature (Cairo et al. 2012; Dwan et al. 2013). Documenting, transparent reporting, and disseminating of methodological limitations and negative outcomes—such as early implant loss, biological complications (e.g., peri‐implantitis), mechanical failures, or unexpected tissue responses—thus contributes to the overall safety and effectiveness of implant therapies. Clinicians may recognize potential risks, refine patient selection criteria, and adjust treatment protocols accordingly. Negative findings not only foster scientific integrity and reproducibility but also contribute directly to evidence‐based risk assessment and patient safety. Moreover, given that many adverse events go unreported in routine practice, peer‐reviewed publications have a critical role in ensuring that such data reach clinicians who may otherwise remain unaware of uncommon but clinically relevant complications.

For future clinical translation, it would be essential to emphasize that zirconia implant systems are not a uniform category. Differences exist between one‐piece and two‐piece designs, bone‐level versus tissue‐level configurations, as well as cemented versus screw‐retained prosthetic options, each carrying distinct clinical implications. Future studies should take these factors into account to provide more tailored recommendations for implant selection and prosthetic protocols in daily practice when using zirconia implants.

## 5 Conclusions

The investigated one‐piece zirconia implant showed a lower survival rate compared to other one‐piece zirconia and two‐piece titanium implants, with frequent bone loss exceeding 2 mm. No direct link to potential confounding factors could be found. The implant's unique tapered design and surface treatment likely contributed to its poor performance. In particular, the rapid surface degradation due to aging may have compromised stable bone integration. Nonetheless, the findings—despite being negative—provide valuable insights into the long‐term clinical behavior of zirconia implants.

## Author Contributions

**Ralf‐Joachim Kohal:** conceptualization, methodology, investigation, funding acquisition, writing – original draft, project administration, resources, supervision. **Agneta Lith:** investigation, writing – review and editing, validation, methodology, software. **Futoshi Komine:** investigation, formal analysis, writing – review and editing. **Junichi Honda:** investigation, formal analysis, writing – review and editing. **Benedikt C. Spies:** investigation, formal analysis, writing – review and editing, conceptualization. **Felix Burkhardt:** writing – original draft, formal analysis, visualization, validation. **Kirstin Vach:** validation, software, writing – review and editing, formal analysis, data curation, conceptualization.

## Conflicts of Interest

Ralf‐Joachim Kohal was a lecturer for Nobel Biocare from 2005 to 2008. Futoshi Komine, Benedikt C. Spies, Agneta Lith, Felix Burkhardt, Kirstin Vach declare no conflicts of interest.

## Data Availability

The data that support the findings of this study are available on request from the corresponding author. The data are not publicly available due to privacy or ethical restrictions.

## References

[clr70049-bib-0001] Al‐Ahmad, A. , M. Wiedmann‐Al‐Ahmad , A. Fackler , et al. 2013. “In Vivo Study of the Initial Bacterial Adhesion on Different Implant Materials.” Archives of Oral Biology 58: 1139–1147. 10.1016/j.archoralbio.2013.04.011. 23694907

[clr70049-bib-0059] Altman, D. G. 1999. Practical Statistics for Medical Research. 1st ed. Chapman & Hall/CRC Press.

[clr70049-bib-0002] Altmann, B. , L. Karygianni , A. Al‐Ahmad , et al. 2017. “Assessment of Novel Long‐Lasting Ceria‐Stabilized Zirconia‐Based Ceramics With Different Surface Topographies as Implant Materials.” Advanced Functional Materials 27: 1702512. 10.1002/adfm.201702512.

[clr70049-bib-0003] Berglundh, T. , G. Armitage , M. G. Araujo , et al. 2018. “Peri‐Implant Diseases and Conditions: Consensus Report of Workgroup 4 of the 2017 World Workshop on the Classification of Periodontal and Peri‐Implant Diseases and Conditions.” Journal of Periodontology 89 Suppl 1: S313–S318. 10.1002/JPER.17-0739. 29926955

[clr70049-bib-0004] Bethke, A. , S. Pieralli , R. J. Kohal , et al. 2020. “Fracture Resistance of Zirconia Oral Implants In Vitro: A Systematic Review and Meta‐Analysis.” Materials (Basel) 13, no. 3: 562. 10.3390/ma13030562. 31991565 PMC7040771

[clr70049-bib-0005] Borie, E. , E. Rosas , R. Freitas de Souza , and F. J. Dias . 2024. “Zirconia Implants: A Brief Review and Surface Analysis of a Lost Implant.” Coatings 14: 995. 10.3390/coatings14080995.

[clr70049-bib-0006] Brunello, G. , N. Rauch , K. Becker , A. R. Hakimi , F. Schwarz , and J. Becker . 2022. “Two‐Piece Zirconia Implants in the Posterior Mandible and Maxilla: A Cohort Study With a Follow‐Up Period of 9 Years.” Clinical Oral Implants Research 33, no. 12: 1233–1244. 10.1111/clr.14005. 36184914

[clr70049-bib-0007] Cairo, F. , I. Sanz , P. Matesanz , M. Nieri , and U. Pagliaro . 2012. “Quality of Reporting of Randomized Clinical Trials in Implant Dentistry. A Systematic Review on Critical Aspects in Design, Outcome Assessment and Clinical Relevance.” Journal of Clinical Periodontology 39 Suppl 12: 81–107. 10.1111/j.1600-051X.2011.01839.x. 22533949

[clr70049-bib-0008] Carra, M. C. , N. Blanc‐Sylvestre , A. Courtet , and P. Bouchard . 2023. “Primordial and Primary Prevention of Peri‐Implant Diseases: A Systematic Review and Meta‐Analysis.” Journal of Clinical Periodontology 50 Suppl 26: 77–112. 10.1111/jcpe.13790. 36807599

[clr70049-bib-0009] Chevalier, J. , and L. Gremillard . 2009. “The Tetragonal‐Monoclinic Transformation in Zirconia: Lessons Learned and Future Trends.” Journal of the American Ceramic Society 92, no. 9: 1901–1920. 10.1111/j.1551-2916.2009.03278.x.

[clr70049-bib-0010] Chrcanovic, B. R. , M. D. Martins , and A. Wennerberg . 2015. “Immediate Placement of Implants Into Infected Sites: A Systematic Review.” Clinical Implant Dentistry and Related Research 17 Suppl 1: e1–e16. 10.1111/cid.12098. 23815434

[clr70049-bib-0011] Colak, S. , and M. S. Demirsoy . 2023. “Retrospective Analysis of Dental Implants Immediately Placed in Extraction Sockets With Periapical Pathology: Immediate Implant Placement in Infected Areas.” BMC Oral Health 23, no. 1: 304. 10.1186/s12903-023-02986-0. 37208620 PMC10197846

[clr70049-bib-0012] Daubert, D. , R. M. Black , V. Chrepa , and G. A. Kotsakis . 2020. “Endodontic Peri‐Implant Defects: A New Disease Entity.” Journal of Endodontics 46, no. 3: 444–448. 10.1016/j.joen.2019.12.002. 31959483

[clr70049-bib-0013] de Beus, J. H. W. , M. S. Cune , J. W. A. Slot , et al. 2024. “A Randomized Clinical Trial on Zirconia Versus Titanium Implants in Maxillary Single Tooth Replacement.” Clinical Oral Implants Research 35, no. 6: 630–640. 10.1111/clr.14258. 38567929

[clr70049-bib-0014] Di Murro, B. , L. Canullo , G. Pompa , C. Di Murro , and P. Papi . 2021. “Prevalence and Treatment of Retrograde Peri‐Implantitis: A Retrospective Cohort Study Covering a 20‐Year Period.” Clinical Oral Investigations 25, no. 7: 4553–4561. 10.1007/s00784-020-03769-5. 33443685 PMC8310488

[clr70049-bib-0015] Dwan, K. , C. Gamble , P. R. Williamson , and J. J. Kirkham . 2013. “Systematic Review of the Empirical Evidence of Study Publication Bias and Outcome Reporting Bias—An Updated Review.” PLoS One 8, no. 7: e66844. 10.1371/journal.pone.0066844. 23861749 PMC3702538

[clr70049-bib-0016] Fage, S. W. , J. Muris , S. S. Jakobsen , and J. P. Thyssen . 2016. “Titanium: A Review on Exposure, Release, Penetration, Allergy, Epidemiology, and Clinical Reactivity.” Contact Dermatitis 74, no. 6: 323–345. 10.1111/cod.12565. 27027398

[clr70049-bib-0017] Fretwurst, T. , K. Nelson , D. P. Tarnow , H. L. Wang , and W. V. Giannobile . 2018. “Is Metal Particle Release Associated With Peri‐Implant Bone Destruction? An Emerging Concept.” Journal of Dental Research 97, no. 3: 259–265. 10.1177/0022034517740560. 29130804

[clr70049-bib-0018] Gandolfi, M. G. , F. Zamparini , G. Iezzi , et al. 2018. “Microchemical and Micromorphologic ESEM‐EDX Analysis of Bone Mineralization at the Thread Interface in Human Dental Implants Retrieved for Mechanical Complications After 2 Months to 17 Years.” International Journal of Periodontics and Restorative Dentistry 38, no. 3: 431–441. 10.11607/prd.3503. 29641634

[clr70049-bib-0019] Gao, Y. , and J. Ma . 2024. “Prevention of Retrograde Peri‐Implantitis Caused by Pulpal/Periapical Lesions in Adjacent Teeth: A Literature Review.” Journal of Dentistry 151: 105434. 10.1016/j.jdent.2024.105434. 39481828

[clr70049-bib-0020] He, X. , F. X. Reichl , S. Milz , et al. 2020. “Titanium and Zirconium Release From Titanium‐ and Zirconia Implants in Mini Pig Maxillae and Their Toxicity In Vitro.” Dental Materials 36, no. 3: 402–412. 10.1016/j.dental.2020.01.013. 31992485

[clr70049-bib-0021] Hjalmarsson, L. , M. Gheisarifar , and T. Jemt . 2016. “A Systematic Review of Survival of Single Implants as Presented in Longitudinal Studies With a Follow‐Up of At Least 10 Years.” European Journal of Oral Implantology 9 Suppl 1: S155–S162. 27314122

[clr70049-bib-0022] Hosoki, M. , K. Nishigawa , Y. Miyamoto , G. Ohe , and Y. Matsuka . 2016. “Allergic Contact Dermatitis Caused by Titanium Screws and Dental Implants.” Journal of Prosthodontic Research 60, no. 3: 213–219. 10.1016/j.jpor.2015.12.004. 26774509

[clr70049-bib-0023] Howe, M. S. , W. Keys , and D. Richards . 2019. “Long‐Term (10‐Year) Dental Implant Survival: A Systematic Review and Sensitivity Meta‐Analysis.” Journal of Dentistry 84: 9–21. 10.1016/j.jdent.2019.03.008. 30904559

[clr70049-bib-0024] Huang, M. , C. Wang , P. Li , H. Lu , A. Li , and S. Xu . 2024. “Role of Immune Dysregulation in Peri‐Implantitis.” Frontiers in Immunology 15: 1466417. 10.3389/fimmu.2024.1466417. 39555067 PMC11563827

[clr70049-bib-0025] Karoussis, I. K. , G. E. Salvi , L. J. Heitz‐Mayfield , U. Brägger , C. H. Hämmerle , and N. P. Lang . 2003. “Long‐Term Implant Prognosis in Patients With and Without a History of Chronic Periodontitis: A 10‐Year Prospective Cohort Study of the ITI Dental Implant System.” Clinical Oral Implants Research 14, no. 3: 329–339. 10.1034/j.1600-0501.000.00934.x. 12755783

[clr70049-bib-0026] Kohal, R. , T. Douillard , C. Sanon , A. Kocjan , and J. Chevalier . 2025. “Signs of In‐Vivo Aging of Zirconia From Explanted Dental Implants With Porous Coating After Several Years in Function.” Acta Biomaterialia 194: 498–513. 10.1016/j.actbio.2025.01.020. 39828074

[clr70049-bib-0027] Kohal, R. J. , and D. K. Dennison . 2020. “Clinical Longevity of Zirconia Implants With the Focus on Biomechanical and Biological Outcome.” Current Oral Health Reports 7: 344–351. 10.1007/s40496-020-00289-9.

[clr70049-bib-0028] Kohal, R. J. , M. Knauf , B. Larsson , H. Sahlin , and F. Butz . 2012. “One‐Piece Zirconia Oral Implants: One‐Year Results From a Prospective Cohort Study. 1. Single Tooth Replacement.” Journal of Clinical Periodontology 39, no. 6: 590–597. 10.1111/j.1600-051X.2012.01876.x. 22519944

[clr70049-bib-0029] Kohal, R. J. , E. Riesterer , K. Vach , et al. 2024. “Fracture Resistance of a Bone‐Level Two‐Piece Zirconia Oral Implant System‐The Influence of Artificial Loading and Hydrothermal Aging.” Journal of Functional Biomaterials 15, no. 5: 122. 10.3390/jfb15050122. 38786633 PMC11122605

[clr70049-bib-0030] Kohal, R. J. , F. S. Schwindling , M. Bächle , and B. C. Spies . 2016. “Peri‐Implant Bone Response to Retrieved Human Zirconia Oral Implants After a 4‐Year Loading Period: A Histologic and Histomorphometric Evaluation of 22 Cases.” Journal of Biomedical Materials Research: Part B, Applied Biomaterials 104, no. 8: 1622–1631. 10.1002/jbm.b.33512. 26332678

[clr70049-bib-0031] Kohal, R. J. , B. C. Spies , A. Bauer , and F. Butz . 2018. “One‐Piece Zirconia Oral Implants for Single‐Tooth Replacement: Three‐Year Results From a Long‐Term Prospective Cohort Study.” Journal of Clinical Periodontology 45, no. 1: 114–124. 10.1111/jcpe.12815. 28902420

[clr70049-bib-0032] Kohal, R. J. , K. Vach , F. Butz , B. C. Spies , S. B. M. Patzelt , and F. Burkhardt . 2023. “One‐Piece Zirconia Oral Implants for the Support of Three‐Unit Fixed Dental Prostheses: Three‐Year Results From a Prospective Case Series.” Journal of Functional Biomaterials 14, no. 1: 45. 10.3390/jfb14010045. 36662092 PMC9864364

[clr70049-bib-0033] Lang, N. P. , T. Berglundh , L. J. Heitz‐Mayfield , B. E. Pjetursson , G. E. Salvi , and M. Sanz . 2004. “Consensus Statements and Recommended Clinical Procedures Regarding Implant Survival and Complications.” International Journal of Oral and Maxillofacial Implants 19 Suppl: 150–154. 15635955

[clr70049-bib-0034] Lee, H. G. , A. Hsu , H. Goto , et al. 2013. “Aggravation of Inflammatory Response by Costimulation With Titanium Particles and Mechanical Perturbations in Osteoblast‐ and Macrophage‐Like Cells.” American Journal of Physiology‐Cell Physiology 304, no. 5: C431–C439. 10.1152/ajpcell.00202.2012. 23255578 PMC3602649

[clr70049-bib-0035] Lekholm, U. , and G. A. Zarb . 1985. “Patient Selection and Preparation.” In Tissue Integrated Prostheses: Osseointegration in Clinical Dentistry, edited by P. I. Brånemark , G. A. Zarb , and T. Albrektsson , 199–209. Quintessence.

[clr70049-bib-0036] Lorenz, J. , N. Giulini , W. Hölscher , A. Schwiertz , F. Schwarz , and R. Sader . 2019. “Prospective Controlled Clinical Study Investigating Long‐Term Clinical Parameters, Patient Satisfaction, and Microbial Contamination of Zirconia Implants.” Clinical Implant Dentistry and Related Research 21, no. 2: 263–271. 10.1111/cid.12720. 30714303

[clr70049-bib-0037] Mohseni, P. , A. Soufi , and B. R. Chrcanovic . 2023. “Clinical Outcomes of Zirconia Implants: A Systematic Review and Meta‐Analysis.” Clinical Oral Investigations 28, no. 1: 15. 10.1007/s00784-023-05401-8. 38135804 PMC10746607

[clr70049-bib-0038] Mombelli, A. , D. Hashim , and N. Cionca . 2018. “What is the Impact of Titanium Particles and Biocorrosion on Implant Survival and Complications? A Critical Review.” Clinical Oral Implants Research 29, no. Suppl 18: 37–53. 10.1111/clr.13305. 30306693

[clr70049-bib-0039] Nelson, K. , B. Hesse , O. Addison , et al. 2020. “Distribution and Chemical Speciation of Exogenous Micro‐ and Nanoparticles in Inflamed Soft Tissue Adjacent to Titanium and Ceramic Dental Implants.” Analytical Chemistry 92, no. 21: 14432–14443. 10.1021/acs.analchem.0c02416. 32970419

[clr70049-bib-0040] Orishko, A. , J. C. Imber , A. Roccuzzo , A. Stahli , and G. E. Salvi . 2024. “Tooth‐ and Implant‐Related Prognostic Factors in Treatment Planning.” Periodontology 2000 95, no. 1: 102–128. 10.1111/prd.12597. 39234949

[clr70049-bib-0041] Östman, P. O. , M. Hellman , T. Albrektsson , and L. Sennerby . 2007. “Direct Loading of Nobel Direct and Nobel Perfect One‐Piece Implants: A 1‐Year Prospective Clinical and Radiographic Study.” Clinical Implant Dentistry and Related Research 18, no. 4: 409–418. 10.1111/j.1600-0501.2007.01346.x. 17501980

[clr70049-bib-0042] Pettersson, M. , P. Kelk , G. N. Belibasakis , D. Bylund , M. Molin Thoren , and A. Johansson . 2017. “Titanium Ions Form Particles That Activate and Execute Interleukin‐1Beta Release From Lipopolysaccharide‐Primed Macrophages.” Journal of Periodontal Research 52, no. 1: 21–32. 10.1111/jre.12364. 26987886 PMC5297875

[clr70049-bib-0043] Piconi, C. , and G. Maccauro . 1999. “Zirconia as a Ceramic Biomaterial.” Biomaterials 20, no. 1: 1–25. 10.1016/s0142-9612(98)00010-6. 9916767

[clr70049-bib-0044] Pieralli, S. , R. J. Kohal , E. Lopez Hernandez , S. Doerken , and B. C. Spies . 2018. “Osseointegration of Zirconia Dental Implants in Animal Investigations: A Systematic Review and Meta‐Analysis.” Dental Materials 34, no. 2: 171–182. 10.1016/j.dental.2017.10.008. 29122237

[clr70049-bib-0045] Prati, C. , F. Zamparini , D. Botticelli , et al. 2020. “The Use of ESEM‐EDX as an Innovative Tool to Analyze the Mineral Structure of Peri‐Implant Human Bone.” Materials (Basel) 13, no. 7: 1671. 10.3390/ma13071671. 32260166 PMC7178284

[clr70049-bib-0046] Romanos, G. , F. Zamparini , A. Spinelli , C. Prati , and M. G. Gandolfi . 2022. “ESEM‐EDX Microanalysis at Bone‐Implant Region on Immediately Loaded Implants Retrieved Postmortem.” International Journal of Oral and Maxillofacial Implants 37, no. 3: e51–e60. 10.11607/jomi.9228. 35727252

[clr70049-bib-0048] Sanz, M. , B. Noguerol , I. Sanz‐Sanchez , et al. 2019. “European Association for Osseointegration Delphi Study on the Trends in Implant Dentistry in Europe for the Year 2030.” Clinical Oral Implants Research 30, no. 5: 476–486. 10.1111/clr.13431. 31033047

[clr70049-bib-0049] Schwarz, F. , J. Derks , A. Monje , and H. L. Wang . 2018. “Peri‐Implantitis.” Journal of Clinical Periodontology 45 Suppl 20: S246–S266. 10.1111/jcpe.12954. 29926484

[clr70049-bib-0050] Sennerby, L. , A. Dasmah , B. Larsson , and M. Iverhed . 2005. “Bone Tissue Responses to Surface‐Modified Zirconia Implants: A Histomorphometric and Removal Torque Study in the Rabbit.” Clinical Implant Dentistry and Related Research 7 Suppl 1: S13–S20. 10.1111/j.1708-8208.2005.tb00070.x. 16137083

[clr70049-bib-0051] Serroni, M. , W. S. Borgnakke , L. Romano , et al. 2024. “History of Periodontitis as a Risk Factor for Implant Failure and Incidence of Peri‐Implantitis: A Systematic Review, Meta‐Analysis, and Trial Sequential Analysis of Prospective Cohort Studies.” Clinical Implant Dentistry and Related Research 26, no. 3: 482–508. 10.1111/cid.13330. 38720611

[clr70049-bib-0052] Sgolastra, F. , A. Petrucci , M. Severino , R. Gatto , and A. Monaco . 2015. “Periodontitis, Implant Loss and Peri‐Implantitis. A Meta‐Analysis.” Clinical Oral Implants Research 26, no. 4: e8–e16. 10.1111/clr.12319. 24382358

[clr70049-bib-0053] Sicilia, A. , S. Cuesta , G. Coma , et al. 2008. “Titanium Allergy in Dental Implant Patients: A Clinical Study on 1500 Consecutive Patients.” Clinical Oral Implants Research 19: 823–835. 10.1111/j.1600-0501.2008.01544.x. 18705814

[clr70049-bib-0054] Spies, B. C. , M. Balmer , R. E. Jung , I. Sailer , K. Vach , and R. J. Kohal . 2019. “All‐Ceramic Single Crowns Supported by Zirconia Implants: 5‐Year Results of a Prospective Multicenter Study.” Clinical Oral Implants Research 30, no. 5: 466–475. 10.1111/clr.13433. 30972828

[clr70049-bib-0055] Spitznagel, F. A. , M. Balmer , D. B. Wiedemeier , R. E. Jung , and P. C. Gierthmuehlen . 2022. “Clinical Outcomes of All‐Ceramic Single Crowns and Fixed Dental Prostheses Supported by Ceramic Implants: A Systematic Review and Meta‐Analyses.” Clinical Oral Implants Research 33, no. 1: 1–20. 10.1111/clr.13871. 34665900 PMC9297865

[clr70049-bib-0056] Stawarczyk, B. , C. Keul , M. Eichberger , D. Figge , D. Edelhoff , and N. Lumkemann . 2017. “Three Generations of Zirconia: From Veneered to Monolithic. Part I.” Quintessence International 48, no. 5: 369–380. 10.3290/j.qi.a38057. 28396886

[clr70049-bib-0057] Tschernitschek, H. , L. Borchers , and W. Geurtsen . 2005. “Nonalloyed Titanium as a Bioinert Metal—A Review.” Quintessence International 36, no. 7–8: 523–530. 15997933

[clr70049-bib-0058] van Steenberghe, D. 1997. “Outcomes and Their Measurement in Clinical Trials of Endosseous Oral Implants.” Annals of Periodontology 2, no. 1: 291–298. 10.1902/annals.1997.2.1.291. 9151562

